# Lactoferrin from Bovine Milk: A Protective Companion for Life

**DOI:** 10.3390/nu12092562

**Published:** 2020-08-24

**Authors:** Fabiana Superti

**Affiliations:** National Centre for Innovative Technologies in Public Health, National Institute of Health, Viale Regina Elena 299, 00161 Rome, Italy; fabiana.superti@iss.it; Tel.: +39-06-4990-3149

**Keywords:** lactoferrin, bovine milk, nutraceutical, human health

## Abstract

Lactoferrin (Lf), an iron-binding multifunctional glycoprotein belonging to the transferrin family, is present in most biological secretions and reaches particularly high concentrations in colostrum and breast milk. A key function of lactoferrin is non-immune defence and it is considered to be a mediator linking innate and adaptive immune responses. Lf from bovine milk (bLf), the main Lf used in human medicine because of its easy availability, has been designated by the United States Food and Drug Administration as a food additive that is generally recognized as safe (GRAS). Among the numerous protective activities exercised by this nutraceutical protein, the most important ones demonstrated after its oral administration are: Antianemic, anti-inflammatory, antimicrobial, immunomodulatory, antioxidant and anticancer activities. All these activities underline the significance in host defence of bLf, which represents an ideal nutraceutical product both for its economic production and for its tolerance after ingestion. The purpose of this review is to summarize the most important beneficial activities demonstrated following the oral administration of bLf, trying to identify potential perspectives on its prophylactic and therapeutic applications in the future.

## 1. Introduction

Lactoferrin, an 80 kDa iron-binding glycoprotein belonging to the family of transferrin proteins, was first isolated in 1939 from cow’s milk [1] and in 1960 was shown to be the main iron-binding protein in human milk [2]. Lactoferrin is also found in mucosal secretions such as tears, saliva, vaginal mucus, seminal plasma, nasal and bronchial secretions, bile, gastrointestinal fluids and urine [3]. It is present in plasma in relatively low concentrations, where it is predominantly neutrophil derived [4].

Bovine lactoferrin (bLf) has been extensively studied in the past 60 years, as research on this protein actually started around the 1960s, when technological progress had allowed its correct extraction from milk and its complete characterization [5].

Its role in numerous and varied biological functions is now accepted by the scientific community. Indeed, it has been shown that bLf is involved in various physiological and protective actions, among which some of the most studied to date are antioxidant, anti-tumour, anti-inflammatory and antimicrobial activities [6,7,8,9,10,11,12,13].

In this review on bLf, both the main characteristics and the major biological functions of this pleiotropic nutraceutical protein will be summarized. In particular, the use of exogenous bLf as a therapeutic agent and the mechanisms responsible for its various actions will be taken into consideration in order to identify new research perspectives.

## 2. Bioavailability, Metabolism, Absorption and Delivery of Bovine Lactoferrin

As previously mentioned, bLf, from milk or whey, is used to improve immunity, resistance to infection, control of non-communicable diseases, iron absorption and human health in general. Since many of these functional properties are highly dependent on the structural integrity of the protein, it must be remembered that when bLf is taken orally it can be largely digested in the stomach [14]. In particular, since bLf receptors are found in the intestinal mucosa and in the cells of the lymphatic tissue of the intestine [15,16], it is important that bLf maintain its structural integrity to bind its receptors. However, it has been shown that bLf directly induces the growth and proliferation of enterocytes, depending on its concentration [17], so intestinal absorption of lactoferrin can be different in different periods of life. It is noteworthy that at the beginning of life the intestinal lumen of the baby who is breastfed or fed with infant formula fortified with bLf will have a high concentration of lactoferrin attributable to very limited proteolytic degradation and high cell proliferation [18]. The mucosal development induced by lactoferrin can, thus, increase the mucosal surface and not only improve the absorption of iron but also of other nutrients. Later, as the baby grows, the digestion of proteins will be more efficient and the lactoferrin concentration will be much lower, resulting in increased differentiation. Hence, in adulthood, as previously mentioned, bLf administered orally will be largely digested into small molecules. Since many functions of bLf (such as the ability to bind iron) are highly dependent on the integrity of the protein structure, its gastrointestinal digestion causes a loss of many of these properties. However, protein degradation also has positive aspects as some peptides produced by its digestion, such as lactoferricin, a 25-residue peptide (Lf amino acid residues 17–41) [19], and lactoferrampin, a 20-residue peptide (Lf amino acid residues 265–284) [20], display potent defensive activity. These peptides possess antimicrobial activity due to their hydrophobicity and cationic charge that make them amphipathic molecules. Lactoferricin that in some cases displays a more potent antibacterial and anti-fungal activity than intact bLf [19,21] possess antimicrobial [22,23,24], anticancer [24,25,26,27] and anti-inflammatory properties [28], while lactoferrampin shows a wide antimicrobial action against bacteria, viruses, yeasts and parasites [22,24]. Finally, it has been reported that lactoferricin, incorporated in food supplements, could provide health benefits and reduce the risk of chronic disease [29]. Additional studies are needed to identify all biological activities (together with the molecular mechanisms involved) of these bioactive peptides derived from the digestion of bLf. This is essential in order to optimize their use for human health and well-being. Further insights into the multiple activities of these two peptides can be found in the reviews of Gifford et al. [24], Bruni et al. [30] and Drago-Serrano et al. [31].

As mentioned above, bLf receptors are found in the intestine [15,16], so the orally administered protein must be protected to pass through the stomach and reach the intestine without being degraded. In order to improve its oral bioavailability, the formulation of bLf oral delivery systems has been approached with different approaches. Among the most commonly used methods to protect bLf during the oral and gastric passage phases we find: Iron saturation, microencapsulation, PEGylation and absorption enhancers [14,32]. While it is believed that iron saturation is one of the methods for slowing the enzymatic hydrolysis of bLf, it is not considered an effective method of delivering bLf in its structurally intact form to small intestine by oral administration [33]. Microencapsulation is a commonly used method to protect bLf from protease digestion. This method involves the formation of a protective structure (protein or polysaccharide shell) around the bLf core. This core-shell system effectively protects the bLf from gastric digestion and, by using appropriate shell materials, can also allow for specific and controlled release of the protein. In addition to microencapsulation with proteins or carbohydrates, liposomes have also been shown to prevent gastric degradation of bLf [34]. PEGylation, i.e., the covalent attachment of polyethylene glycol (PEG) to therapeutic proteins, is used to protect bLf from the gastric environment. This technique increases bLf resistance to proteases through steric hindrance and, by increasing molecular mass, inhibits renal clearance [32]. As for absorption stimulators, these are a group of chemicals that increase the permeability or transport of molecules across biological membranes. In the field of bLf, research on absorption stimulators focused on chitosan, a linear polysaccharide composed of randomly distributed beta-(1->4)-linked D-glucosamine (deacetylated unit) and N-acetyl-D-glucosamine (acetylated unit). Chitosan has been reported to increase bLf uptake in the gut by opening the intercellular junctions [35]. However, chitosan tends to dissolve at acidic gastric pH, so, to overcome this problem, chitosan derivatives which are poorly soluble in acidic conditions, such as chitosan-succinate and chitosan-phthalate, have been used [32]. Therefore, regarding the oral bioavailability of bLf, we can conclude that, at present, microencapsulation and PEGylation appear to be the most efficient methods to deliver bLf to gut absorption sites.

## 3. Lactoferrin, Iron, Oxidative Stress and Anaemia

**Figure 1:**
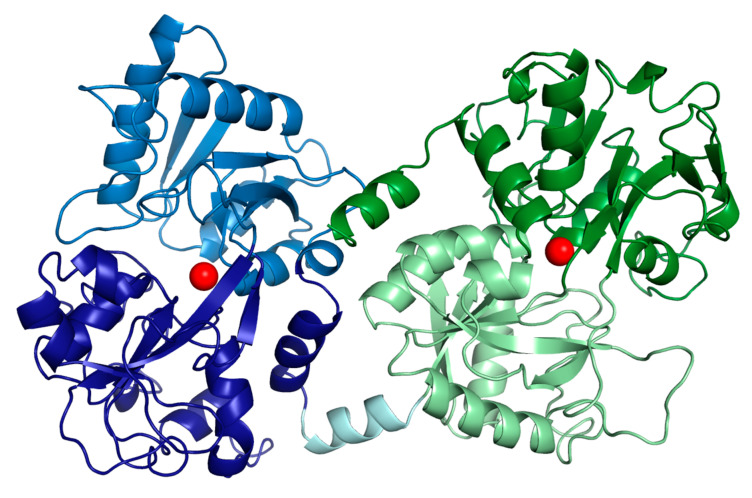
Cartoon representation of bovine lactoferrin. N-lobe is blue (N1 pale blue and N2 dark blue) and C-lobe is green (C1 dark green and C2 pale green). The hinge helix is represented in a pale cyan. Iron ions are reported as red spheres.

Lactoferrin, as the other transferrins, has a molecular weight of about 80 kDa and its structure includes two lobes, each capable of reversibly chelating two Fe^+3^ ions per molecule. Both lobes have the same fold, consistent with their sequence identity of ~40%. In each lobe, two domains, referred to as N1 and N2, or C1 and C2, enclose a deep fissure containing the conserved iron-binding site [36]. It is usually only about 15% saturated with iron, indicating that the two lobes are not entirely occupied by iron. bLf, possessing twice the serum transferrin’s affinity for iron, is also able to act on systemic iron homeostasis by modulating the synthesis of the two key proteins hepcidin and ferroportin through the down-regulation of interleukin-6 (IL-6) [37]. Figure 1 shows the two Fe^+3^ binding domains of bLf.

### 3.1. bLf Protection against Iron Deregulation and Oxidative Stress

Iron, an essential nutrient for cell growth, can become toxic when too abundant, leading to the generation of free radicals by interconverting between its most common oxidative forms, ferrous (Fe^2+^) and ferric (Fe^3+^) forms [38]. Free iron is toxic because it can donate or receive an electron from adjacent molecules, causing damage to cellular components or generating reactive oxygen species (ROS) that are themselves cytotoxic.

bLf controls the physiological balance of ROS production and their elimination rate through iron sequestration. Many researchers have demonstrated that bLf is able to modulate the adaptive immune system, and that it possesses significant regulation activity on cellular redox via upregulation of key antioxidant enzymes [39,40,41,42]. Oxidative stress plays a role in numerous chronic degenerative processes, such as those that affect tumour development, inflammation and aging [12]. Notwithstanding factors responsible for the ROS production imbalance having not been fully elucidated, it is known that the rate and extent of ROS development removal is dependent on the efficiency of superoxide dismutase (SOD), glutathione peroxidase (GPx) and catalase (CAT). SOD converts the superoxide radical (•O_2_^−^) into hydrogen peroxide (H_2_O_2_); GPx or CAT transform H_2_O_2_ into water (H_2_O) or into H_2_O and molecular oxygen (O_2_), respectively. In the presence of free ferric ions (Fe^3+^) the superoxide radical (•O_2_^−^) can be degraded through two phases: In the first, a superoxide molecule reacts with Fe^3+^ to form ferrous ion (Fe^2+^) and O_2_; in the second (Fenton reaction), Fe^2+^ reacts with H_2_O_2_ to form Fe^3+^, a hydroxyl radical (•OH) and a hydroxide ion (OH^−^). The reaction of the hydroxyl radical with polyunsaturated fatty acids, causing the removal of a hydrogen atom, starts the lipid peroxidation and the production of new radicals.

**Figure 2:**
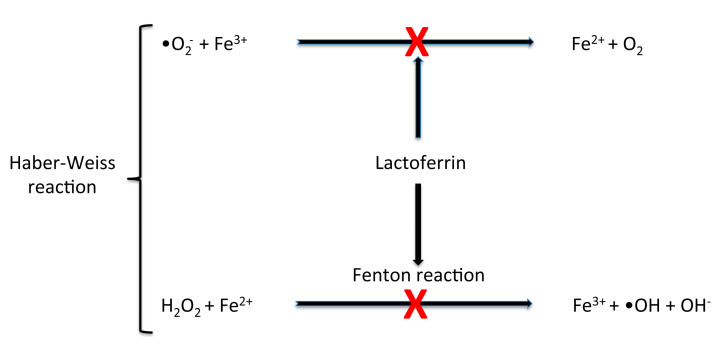
Lactoferrin protects against cellular damage induced by oxidative stress. It inhibits free ferric ion reactivity with superoxide molecules, thus limiting the formation of ferrous salt and ground state oxygen. This effect prevents the Fenton reaction in which the ferrous ion is oxidized by hydrogen peroxide to ferric ion, forming a hydroxyl radical and a hydroxide ion.

bLf by sequestering Fe^3+^, is able to prevent the harmful effects of oxidative stress and many studies have demonstrated that it contributes to general homeostasis by disrupting the production of these dangerous radicals [12]. Figure 2 shows Fenton and Haber–Weiss reactions.

An example of these studies on bLf protection against iron deregulation and oxidative stress is the one conducted by Okazaki et al. [43] that examined the antioxidant property of bLf oral administration in a rat model of ferric nitrilotriacetate-induced renal tubular oxidative injury. Results of this research showed that bLf pre-treatment suppressed elevation of either serum creatinine or blood urea nitrogen levels and exerted protective effects against renal oxidative tubular damage. These results not only demonstrated the antioxidative effect of bLf but also indicate that lactoferrin consumption is useful in the prevention of iron-mediated renal tubular oxidative damage [43].

### 3.2. bLf in the Prevention and Treatment of Iron Deficiency Anaemia

Homeostasis is the maintenance of balance in a biological system and is controlled by several factors including bLf which, for its ability to bind ferric ions, plays a central role. Indeed, iron homeostasis is regulated in part by bLf which plays a safety role by protecting against oxidative stress and reducing the amount of cell damage induced by insult. An increasing amount of data have shown the association between disruption of iron homeostasis and different pathophysiologic conditions such as anaemia and, in particular, Fe-overload related disorders [44].

Anaemia, defined as number of red blood cells or haemoglobin concentration below established cut-off levels [45], is a worldwide disease that should not be underestimated as it has important consequences for human health. Its prevalence in pre-school aged children, pregnant women and women of reproductive age is approximately 50%, 40% and 30%, respectively [46]. WHO has estimated that about 50% of all cases of anaemia can be attributed to iron deficiency [47]. It is well known that iron, an essential component of haemoglobin, is found both in plant and animal foods but it is better absorbed from animal sources [48]. In this view, lactoferrin, being one of the main iron-binding proteins also responsible for its transport and release into cells, represents a key element of the iron absorption process.

On the basis of these considerations, several studies have been carried out to evaluate the efficacy of oral administration of bLf for the treatment of iron deficiency anaemia (IDA). In these researches the effectiveness of bLf has often been compared to that of ferrous iron preparations, sometimes leading to partially conflicting results.

Fransson et al. in 1983 [49] analysed the efficacy of bLf supplementation in iron-deficient and iron-sufficient young mice demonstrating that this transferrin represents a useful vehicle for iron supplementation. In this research, the efficacy of lactoferrin supplementation was compared with that of iron chloride and no significant differences were observed. Successively, as IDA during pregnancy represents a risk factor for preterm delivery, the effect of bLf supplementation was studied in women at different trimesters of pregnancy and compared with that of ferrous sulphate. Unlike what was previously observed in mice, in this study haemoglobin and total serum iron values increased to a greater extent in women treated with bLf compared to those who received ferrous sulphate [50]. In a subsequent clinical study by the same authors on pregnant women with IDA, it has been demonstrated that the number of red blood cells, haemoglobin and serum iron increased when they received bLf and decreased when they received ferrous sulphate [51]. In particular, during bLf therapy, serum IL-6 concentrations decreased. So, since IL-6 induces hypoferremia and causes anaemia, bLf is likely to improve serum iron and haemoglobin concentrations, rather than providing more absorbable iron [9]. These results of Paesano et al. [51] are partially in disagreement with those obtained by Nappi et al. [52] who, in a prospective, randomized, controlled, double blind trial, compared the effects of bLf with ferrous sulphate on iron nutritional status in 100 pregnant women with IDA. In fact, the results of this trial showed that bLf and ferrous sulphate had the same efficacy in restoring iron deposits. However, it is important to note that bLf had significantly lower gastrointestinal side effects than ferrous sulphate. Recently, a systematic review and meta-analysis performed to evaluate the efficacy of daily oral bLf compared to daily oral ferrous iron preparations for the treatment of IDA in pregnancy confirmed results reported above, suggesting lactoferrin as the iron replacement agent of choice for IDA treatment in pregnancy [53].

Anaemia is also often observed in endurance athletes (sports anaemia). Especially, female long distance runners, who menstruate and accurately control their weight, can easily develop this type of anaemia. Hence, Koikawa et al. [54] conducted a study to verify whether taking bLf could improve or prevent anaemia in these athletes. The results of this study have shown that bLf increases iron absorption among female long distance runners, suggesting that it can be helpful in preventing sports anaemia.

Concerning pre-school aged children, a prospective, multicentre, controlled intervention study on 260 infants (ages 4 to 6 months) evaluated and compared the effect of an iron-fortified formula containing bLf and an iron-fortified formula without bLf on hematologic indexes and iron status in term infants [55]. Results of this study demonstrated that significant increases in total body iron content and iron absorption in the intestine were observed only in infants fed with lactoferrin fortified formula milk. Further information on the role of lactoferrin in the fight against iron deficiency and IDA in newborns and infants are available in the reviews of Ochoa et al. [56] and Cerami [57].

## 4. Lactoferrin in the Defences of the Babies: Decreased Risk of Sepsis and Necrotizing Enterocolitis in Preterm Infants

As just mentioned at the end of the previous paragraph, lactoferrin is fundamental in the infant’s diet. It is important to note that lactoferrin also plays important functions both in protecting the newborn infants from infections and in promoting the maturation of their innate and adaptive immune system. In fact, term and, in particular, preterm infants are at risk of infections. In preterm neonates, necrotizing enterocolitis (NEC), a destructive inflammatory bowel condition and sepsis are causes of severe morbidity and represent the most common motives of death in the first weeks of life and breastfeeding is known to reduce the risks of these serious conditions.

Based on the numerous activities of bLf, in particular the antimicrobial, antioxidant and anti-inflammatory ones, some of which will be better described later, and on the observation that bLf is well tolerated, several clinical studies were conducted that examined the usefulness of the administration of lactoferrin (in general commercial bLf added to infant formula) in the prevention of infections in preterm and term neonates as well as in the reduction of mortality or major morbidity [58,59,60,61,62,63,64]. Results of these clinical trials are summarized in Table 1.

These clinical studies are particularly interesting, not only because they were targeted to the critical VLBW infants, but above all because both mortality and morbidity following sepsis and NEC remain high despite the use of powerful antimicrobial agents [65]. The results of these trials have shown that the administration of bLf in preterm infants, in the absence or in the presence of the probiotic LGG strain, was able to reduce blood infection without adverse effects.

While these results are extremely encouraging, studies are still needed to establish more precisely the dosage, duration of treatment and development of premature babies.

The data obtained so far support the usefulness of further examining the effects of bLf supplementation on the immune response, in particular to infections, in highly vulnerable infants. It is hoped that the results of the numerous on-going studies will definitively demonstrate the benefits of integrating bLf into the preterm baby’s diet leading the way the use of bLf in a clinical setting. More insights into the role of lactoferrin in neonatology can be found in the review by Sharma et al. [66].

## 5. Antimicrobial Activity of bLf

The antimicrobial effect was the first identified lactoferrin protective activity and has been widely demonstrated both in vitro and in vivo [8,10,67]. The bacteriostatic and bactericidal activity of lactoferrin against a large number of gram-positive and gram-negative bacteria is due to two distinct mechanisms [8,10,67,68]. bLf primary role involves the binding and sequestration of free iron at the infection sites, thus depriving microorganisms of this essential substrate for their growth and inducing a bacteriostatic effect [36]. Differently, bactericidal activity is independent of iron and involves direct interaction with the infectious agent: Specific interactions have been described both with lipoteichoic acid (LTA) of gram-positive bacteria and with lipopolysaccharide (LPS) of gram-negative bacteria [67]. Iron sequestration by bLf also prevents biofilm formation that represents a crucial step in the development and persistence of infection [69].

Further mechanisms of the antimicrobial action of bLf are: Rupture of the cell membrane of pathogens, proteolysis of microbial virulence factors, inhibition of microbial adhesion to host cells by binding with glycosaminoglycans (GAGs) and improvement of the growth of normal commensal probiotic microflora in the intestine [67,70].

Concerning in vivo preclinical studies, twenty years ago Wada et al. [71] demonstrated in germfree BALB/c mice that the administration of 10 mg bLf for 3–4 weeks significantly reduced the number of *Helicobacter pylori* in the stomach and also inhibited the attachment of bacteria to it. Numerous in vivo studies have been conducted since then, many of which are described in the review of Teraguchi et al. [72]. The satisfactory results obtained in animal models then led to clinical trials. For example in 2005 Okuda et al. [73] confirmed the activity of bLf in inhibiting colonization by *Helicobacter pylori* in humans. In this double-blind placebo-controlled randomized trial, healthy subjects positive for *Helicobacter pylori* received bLf tablets (200 mg/day) or placebo tablets for 12 weeks. After treatments the decrease of the (13) C-urea breath test values in the bLf group was significantly higher than that in the control group suggesting that bLf administration is effective to suppress *Helicobacter pylori* colonization. *Helicobacter pylori* infection, still very frequent, causes chronic active gastritis and can have serious complications such as gastric malignancies. Since antibiotic treatment (mainly clarithromycin and levofloxacin) has led to an increase in antibiotic-resistant strains in recent decades, these results are of particular interest for the development of a new eradication therapy. This represents only one example of the applications of bLf as an antimicrobial agent in humans since many other studies have shown that oral administration of bLf can reduce bacterial and fungal infections mainly in the gastrointestinal tract [74].

Among the many activities carried out by bLf to fight infections, it should be remembered that bLf also acts as a prebiotic by promoting the growth of beneficial bacteria for the host such as probiotics. So, concerning in vivo preclinical and clinical studies, there are a number of experimental observations that oral administration of bLf, alone or in association with probiotic strains, is able to counteract bacterial and fungal vaginal infections [70].

The antiviral activity of bLf has been extensively studied in in vitro systems [67,75,76] and two main mechanisms have been identified by which bLf inhibits viral infection: (i) Competition with the virus for the binding to cell receptors [77,78]; (ii) direct interaction with capsid or viral envelope proteins [67,75,76,79]. An in vivo preclinical study by Shin et al. [80] demonstrated that orally administered bLf reduced pneumonia in mice infected with the Influenza virus by suppressing the infiltration of inflammatory cells in the lung.

Concerning the effects of lactoferrin oral administration against viral infections in humans, its beneficial action has been demonstrated for different viruses such as hepatitis C virus (HCV) [81,82], rotavirus [83], norovirus [84] and common cold infections [85]. Very recently, clinical use of liposomal bLf in seventy-five patients affected by SARS-CoV-2 infection has been reported [86]. The use of liposomes arises from the observation that liposomes loaded with bLf improved the resistance of bLf to digestive enzymes thus enhancing the effect of orally administered bLf [87]. All 75 COVID-19 positive patients were successfully treated with the oral administration of liposomal bLf, which allowed a complete and fast recovery. As aerosol liposomal therapy is widely employed with good results [88,89], in some patients with headache, dry cough and nasal congestions liposomal bLf was also administered by aerosol that was very useful to relieve not only the respiratory symptoms but also the cough, the headache and the smell and taste dysfunction. The results of this study are very encouraging as they indicated that oral treatment with liposomal bLf induces a fast recovery in 100% of patients and that lower dose of the same treatment (half doses) seems to exert a potential preventive effect against COVID-19 in healthy family members in direct contact with the affected patients [86]. The use of bLf trapped in liposomes will be better discussed in the section on the anticancer activity of bLf. Given the emergence of containing this terrible pandemic, further studies are underway on the use of different forms of Lf to treat COVID-19 patients.

Regarding the antifungal activity of bLf, most of the studies involved *Candida albicans,* known as one of the most dangerous opportunistic pathogens. As for bacteria, bLf can act effectively on a broad spectrum of fungal species due to its strong iron-absorbing property. It has been shown that bLf is capable of killing *Candida albicans* [90]. However, in addition to the iron-depriving effect, bLf is able to directly bind the surface of fungal cells, resulting in increased membrane permeability and inducing their death. The combination of bLf with other antifungal compounds (such as fluconazole) significantly enhanced the inhibitory activity against *Candida albicans* [91] and *Cryptococcus neoformans* [92]. Concerning in vivo studies, it has been reported that, in guinea pigs infected with *Trichophyton mentagrophytes*, orally administered bLf did not prevent development of symptoms during the early phase of infection, but facilitated clinical improvement of skin lesions after the peak of the symptoms [93]. These results indicate the potential utility of bLf as a food component to promote the treatment of dermatophytosis. Other authors developed an experimental model of reproducible oral candidiasis, with immunosuppressed mice, showing local symptoms characteristic of oral thrush in humans and, using this model, demonstrated the efficacy of bLf against experimental *Candida albicans* oral infection [94]. For further information see also the reviews from Superti and De Seta [70] and Fernandes and Carter [95].

In summary, numerous in vivo studies have shown that oral administration of bLf is able to counteract various bacterial, viral and fungal infections. With regard to communicable diseases in general, it is important to remember that, due to the frequent use of antimicrobial drugs, numerous pathogens have become prone to drug resistance which represents the main cause of the unsatisfactory results of some conventional antimicrobial treatments. Consequently, research and development of new therapeutic have become urgent. From this point of view, bLf can represent a very promising tool as an alternative or complementary therapeutic approach to conventional therapy.

## 6. Anti-Inflammatory Activity of bLf

Inflammation is a complex pathophysiological process involving numerous mediators and various cell types in response to microbial or non-microbial injury [12]. If inflammation is not promptly limited, it can cause damage to the host by establishing systemic and even chronic inflammatory conditions. It is well known that the production of principal immune mediators, such as cytokines and chemokines, depends on the recruitment of inflammatory cells and, in particular, innate immune cells.

Several studies demonstrated that lactoferrin, being a natural immunomodulator, exerts an anti-inflammatory effect [96] supported by the strong increase of its content in body secretions during inflammation [97,98]. There are numerous evidences concerning the capability of lactoferrin to improve injury induced by insult and protect the integrity of organs during the development of inflammation. The anti-inflammatory activity of bLf can be partially ascribed to its positive charge through which it interacts with negatively charged groups (for example proteoglycans) present on the surface of the immune cells. This interaction can activate signalling pathways that induce a physiological anti-inflammatory reaction [41]. bLf is also able to enter cells and translocate to the nucleus [99], so regulating pro-inflammatory gene expression [100]. The anti-inflammatory effect of lactoferrin during bacterial infection is also due to its ability to neutralize negatively charged microbial molecules such as LPS, thus preventing the interaction of the LPS-binding protein with the endotoxin and blocking the binding of LPS with the membrane protein CD14 and the subsequent activation of monocytes and macrophages [101].

It is also likely that lactoferrin controls inflammatory response by preventing iron-mediated free radical injury at inflamed sites [9] so, through the control of oxidative stress, it modulates innate immune responsiveness that alters production of immune regulatory mediators that are important for directing development of adaptive immune function [39,102]. Several mechanisms are involved in the immunomodulating activity of lactoferrin [103,104]. Lactoferrin acts on B cells to allow their successive interaction with T cells, promotes the maturation of T cell precursors into T helper cells and induces the differentiation of immature B cells into antigen presenting cells [103]. It has been also suggested that lactoferrin may play a role in T cell activation through modulation of dendritic cell function [105]. The anti-inflammatory effect is probably due to the inhibition of production of proinflammatory cytokines such as interleukin-1 beta (IL-1 beta), IL-6 and TNF-alpha. This, as mentioned before, can be obtained by the translocation of lactoferrin to the nucleus, where it blocks NF-kB (nuclear factor kappa-light-chain-enhancer of activated B cells) activation. It has long been known that bLf is able to limit irritation both at the level of the skin and within the subcutaneous tissues and internal organs and many studies on the immunomodulatory effects of orally administered bLf have been carried out [106]. For further information see also the reviews from Kruzel et al. [12] and Drago-Serrano et al. [107].

### 6.1. Lactoferrin and Dermatitis

Allergic contact dermatitis is an inflammation of the skin resulting from exposure to irritants and allergens present in the environment. The main therapeutic approaches to limit the symptoms of skin allergies include the use of topical corticosteroids and calcineurin inhibitors, which have side effects [108]. Consequently, to overcome the limitations of the currently available treatments, new therapeutic categories including biological ones were considered [109]. In this view, Zimecki et al. [110] carried out a study in BALB/c mice to compare the immunomodulatory actions of bLf on the elicitation phases of the cellular and humoral cutaneous immune responses to oxazolone and toluene diisocyanate, respectively. This study showed that bLf is able to differentially influence the stimulation phases of humoral and cellular immune responses in mouse skin models and that the inhibition of the cellular immune response is probably due to the suppression of Th1 cells.

### 6.2. Lactoferrin and Inflammatory Bowel Diseases

Togawa et al. [111] examined the potential ability of bLf to attenuate colitis utilising a 2,4,6-trinitrobenzenesulfonic acid (TNBS)-induced colitis model in rats. This is a well-established model very similar to human inflammatory bowel disease characterized by mucosal infiltration of neutrophils mediated, at least in part, by tumour necrosis factor-alpha (TNF-alpha) and IL-1beta activation [112]. Results obtained showed that bLf administration is able to suppress the activation of proinflammatory cytokines, such as TNF-alpha, IL-1beta and IL-6 in rats with TNBS-induced colitis. Similar results have been obtained by the same research group in a dextran sulphate sodium (DSS) induced-colitis rat model [113]. The ability of bLf to relieve the inflammatory conditions of DSS-induced experimental colitis was later confirmed in BALB/c mice as well [114]. Since, as expected, iron-free bLf (apo-bLf) treatment was better than iron saturated bLf (holo-bLf) treatment, the results of this study suggested therapy with apo-bLf as a helpful tool in clinical management of ulcerative colitis. More recently, it has been demonstrated in in vitro and ex vivo systems that bLf markedly inhibited expression of pro-inflammatory cytokines, such as TNF-alpha, interleukin-8 (IL-8) and IL-6, both in cultured and Crohn-derived intestinal cells [100]. Investigating the dose-dependent effects of bLf, it has been also observed that it is able to modulate neonatal intestinal inflammation [115]. In this study, the effects of bLf at doses comparable to the levels of lactoferrin in bovine and human milk were analysed using intestinal epithelial cells, as the in vitro system, and immature pig intestine, as the in vivo system. Results obtained demonstrated beneficial effects of bLf at low doses (0.1–1 g/L, close to its levels in cow and human milk, respectively) and harmful effects at a high dose (10 g/L, close to hLf levels in colostrum). These researches, demonstrating that moderate doses of bLf increase the proliferation of intestinal cells while high doses trigger inflammation, are fundamental for establishing effective doses of bLf for the integration of formula in preterm infants, in order to support intestinal maturation and prevent inflammation. This study has important biological significance because it shows that bLf does not always have a beneficial effect but, at high doses and under certain conditions, it can exert a proinflammatory effect.

### 6.3. Lactoferrin and Pulmonary Inflammation Disorders

Over the past decades, asthma and allergic lung inflammation diseases have become increasingly common. Asthma is a long-term inflammatory disease of the lungs characterized by airway eosinophilia, mucin secretion, IgE production and airway hyperresponsiveness.

In bronchial asthma, oxidative stress exacerbates airway inflammation by inducing different proinflammatory mediators, enhancing bronchial hyperresponsiveness, stimulating bronchospasm and increasing mucin production. Oxidative stress is a consequence of enhanced ROS production by eosinophils recruited into the lungs during exposure to pro-oxidant environmental molecules or to respiratory viruses [116]. It has been demonstrated that ROS generated by reduced nicotinamide adenine dinucleotide phosphate (NADPH) oxidase from environmental molecules, such as pollen grains or their extracts, provide a signal that enhances antigen-induced allergic airway inflammation in mouse [117]. Successively, it has been shown that bLf, as an iron-binding protein, is able to reduce pollen extract-induced airway inflammation [118]. It is interesting to note that apo-bLf, but not holo-bLf, significantly reduced the accumulation of inflammatory cells and the formation of mucin-producing cells in the inflamed respiratory tract of mice.

Zimecki et al. [119] studied the efficacy of both bLf and human lactoferrin (hLf) to decrease allergen (ovalbumin)-induced pleurisy in BALB/c mice. bLf was given either orally or was administered by gavage intragastrically or by an intraperitoneal injection. The results demonstrated the efficacy of Lfs, bLf more than hLf, in reducing pleurisy in a well-established experimental mouse model of ovalbumin-induced pleurisy. This study is of particular interest as it has increased knowledge of the suppressive efficacy of bLf in allergy, suggesting that oral administration of bLf may be effective in improving allergy symptoms in patients.

bLf has also been used successfully in a cystic fibrosis (CF) mouse model [120]. CF is a multifactorial genetic disease that affects several organs, including the respiratory tract, in which iron imbalance, inflammation as well as bacterial infection, play an important role in the chronicity and gravity of lung disease. Results of this study demonstrated that aerosolized bLf was able to reduce infiltrated leukocytes in CF mice and pulmonary iron overload in both control and CF mice. Above all, a significant reduction was observed in ferroportin (the iron-regulated transporter 1), ferritin (the intracellular protein that stores and releases iron in a regulated manner) and in the luminal iron content.

### 6.4. Lactoferrin and Hepatitis

Orally ingested bLf has been shown to provide a wide range of benefits in animal models with inflamed liver [121,122] and clinical use of bLf has also produced several promising outcomes, such as the inhibition of hepatic inflammation in chronic hepatitis C (CHC) patients [81,123].

Concerning in vivo studies, Tsubota et al. [121] utilized Long–Evans Cinnamon rats, which spontaneously develop fulminant-like hepatitis, to evaluate the effect of oral administration of bLf on oxidative liver damage. This study showed that bLf allows the recovery of the reduced base excision repair capacity and reduces the accumulation levels of 8-hydroxy-20-deoxyguanosine (a reliable marker of ROS-induced DNA modifications) and mutations in hepatic mitochondrial DNA, possibly thereby protecting Long–Evans Cinnamon rats from lethal hepatic insufficiency. Based on these observations, it has been suggested that bLf could potentially be useful for the treatment of inflammatory liver diseases induced by oxidative stress.

Successively Kuhara et al. [122] utilized four mouse models of hepatitis induced by D-galactosamine, carbon tetrachloride, D-galactosamine plus lipopolysaccharide and zymosan plus lipopolysaccharide to evaluate the efficacy of oral administration of bLf against hepatitis and to identify its mechanism. Results of this research demonstrated that bLf is able to improve the expression of interleukin 11 (IL-11) and bone morphogenetic protein 2 in the small intestine and to protect mice with hepatitis against inflammation.

Regarding clinical trials, Tanaka et al. [81] carried out a first pilot clinical study demonstrating that lactoferrin could be one potential candidate as an anti-HCV reagent that may be effective for the treatment of CHC patients with low serum concentrations of HCV RNA. Finally, Konishi et al. [123] evaluated the effect of bLf on lipid peroxidation, hepatic inflammation and iron metabolism in patients with CHC. Results of this clinical trial demonstrated that bLf therapy allows improvement in lipid peroxidation and alanine aminotransferase (ALT) levels suggesting its oral administration as a promising therapeutic approach for suppressing oxidative stress and inflammation in patients with CHC non-responders to antiviral therapy.

In conclusion, bLf performs its anti-inflammatory action through different cellular receptors and the activation of various cellular signalling pathways, often via iron-dependent mechanisms. Indeed, its ability to sequester iron and to inhibit ROS formation is a key factor in reducing the damage caused to excessive inflammatory responses. The interaction of bLf with its receptors can trigger several protective effects due to the regulation of enzymatic activities and ROS production, the modification of cell phenotype and cytokine profile, the binding to LPS or the competition with its receptors and the prevention of apoptosis.

## 7. Anticancer Activity of bLf

The World Health Organization [124] reported that, in 2018, 18.1 million people around the world had cancer, 9.6 million cancer patients died and cancer was the cause of about 30% of all premature deaths from non-communicable diseases (NCDs) among adults aged 30–69. So the incidence of cancer is getting higher and there is still no fully efficacious cure for all different forms of the disease. Therefore, preventing the development of carcinomas and treating them is critical to reduce current cancer mortality.

The anti-tumour activity of hLf and bLf has been extensively studied for both prevention and treatment, and several mechanisms have been suggested such as intra- and extra-cellular effects or immunoregulatory and anti-inflammatory functions.

In vitro studies showed that the intracellular effects are generally associated with the arrest of tumour cell growth, while the extracellular ones are mainly related to the interaction between bLf and cell membranes, and the immunoregulatory action of bLf is obtained through the activation of the cells of the immune system that release tumour cytotoxic effectors [11].

Numerous in vivo studies have provided evidence that oral administration of bLf is effective in reducing the development of chemically induced tumours [125,126,127,128,129]. The chemopreventive anticancer effects are probably due to the multiple functions of bLf and, in particular, to the stimulation of the immune response, to the modulation of the carcinogenic metabolic enzymes [127], to the antioxidant activity [129], the induction of cell death in tumour tissue and to the inhibition of angiogenesis [128,130]. Regulation of the immune system is a key factor in the action of bLf against cancer [11] and both innate and adaptive immunity are involved in immunostimulation induced by bLf [131,132,133,134].

It has been demonstrated that orally administered bLf exhibits high bioavailability and selectivity towards tumour cells by inhibiting tumour proliferation, survival, migration, invasion and metastasis [131,135,136,137,138,139]. It is important to underline that bLf is able to promote or inhibit cell proliferation by acting selectively on normal or cancerous cells, respectively [139]. The first study on the suppressive effect of bLf in rat carcinogenesis was carried out by Sekine et al. [125]. These authors demonstrated in male F344 rats treated with azoxymethane that oral administration of bLf (diet containing 2 or 0.2% bLf) induced a significant reduction in the incidence and in the number of adenocarcinomas of the large intestine. Results of this study suggested that bLf might be a promising chemopreventor of colon carcinogenesis. In 1999 Igo et al. [136] examined the effects on tumor growth and metastasis of bLf administered orally to BALB/c mice bearing subcutaneous implants of the highly metastatic colon carcinoma 26. Results of this study showed that bLf demonstrated significant inhibition of lung metastatic colony formation from subcutaneous implanted tumours without appreciable effects on tumor growth. Subsequently, Kuhara et al. [131] investigated the effects of oral administration of bLf on the lung colonization by the same colon carcinoma 26. In this study bLf was efficacious before and after tumor implantation, demonstrating a significant inhibitory effect on experimental metastasis. bLf oral administration increased CD4+ and CD8+ cells in the spleen and peripheral blood and enhanced their cytotoxic activity against colon carcinoma 26. Morevoer, bLf induced an increase of CD4+ and CD8+ cells and of interleukin-18 production in the small intestinal epithelium. The results of this study indicate that the inhibition of metastases by oral administration of bLF could be due to an increase in cellular immunity, probably mediated by the increase in IL-18 production in the intestinal epithelium. As previously described, in addition to modulating cellular immunity, bLf carries out anti-inflammatory activity by eliminating ROS, pro-oxidant agents capable of contributing to the development of cancer. bLf protects the host from ROS-mediated cell and tissue damage by both binding free iron and regulating key antioxidant enzymes [39,40,41,42,43]. In this regard, a recent study has shown in a mouse model of hepatocarcinogenesis induced by diethylnitrosamine that oral treatment with bLf, by inhibiting in a dose-dependent manner the elevation in serum markers of liver carcinoma and inflammation, induces a significant improvement in hepatic histological structures [138]. This study demonstrated that bLf is effective in inhibiting the oncogenic activity of diethylnitrosamine in a mouse model of hepatocarcinogenesis through its ability to alleviate the hepatic inflammation and apoptosis. As regard the selectivity of bLF towards transformed cells, Chea et al. [137] demonstrated, in oral squamous cell carcinoma cell lines, that bLf is able to reverse programming of epithelial-to-mesenchymal transition (a biological process of invasion and metastasis in cancers) to mesenchymal-to-epithelial transition and observed in vivo both inhibition of tumor cell infiltration and increased E-cadherin expression in xenografts of mice administered orally with bLf.

Since one of the desired properties of an ideal anticancer drug is the ability to selectively target transformed cancer cells, an appropriate delivery system can be extremely useful in releasing bLf into the tumour site. From this point of view, liposomes represent an efficient drug delivery system that can significantly improve the therapeutic potential of the encapsulated compounds. For instance, apo-bLf trapped in positively charged liposomes composed of phosphatidylcholine, dioleoyl phosphatidylethanolamine, cholesterol and stearylamine (ratio 6:1:2:1 M) has been shown to have a greater capacity, compared to protein alone, to inhibit the growth of B16-F10 melanoma cells [140]. In addition, it has been demonstrated, in a brain-targeted chemotherapeutical delivery system, that doxorubicin (DOX)-loaded bLf-modified procationic liposome (PCL), effectively improved both uptake and cytotoxicity of bLf against the glioma C6 cell proliferation, as well as the anti-glioma activity in vivo, compared with DOX solution or DOX-loaded conventional liposomes [141]. In this study, a cholesterol derivative (CHETA, C36H61N3O4S2) was used to prepare negatively charged PCLs and, subsequently, bLf (positively charged at physiological pH) was absorbed onto their surface via electrostatic interaction. This study showed that DOX-Lf-PCLs delivery system was effective and feasible for systemic administration in chemotherapy of glioma. These results confirmed and supported previous researches of the same authors in which this drug carrier for brain delivery, PCLs, was evaluated both in vitro and in vivo. In this study an in vitro model of the blood–brain barrier was developed to assess the ability and mechanisms of PCLs and Lf-PCLs to cross endothelial cells whereas the uptake of PCLs and Lf-PCLs by the mouse brain in vivo was detected by HPLC-fluorescence analysis. Results obtained demonstrated that, compared with the conventional liposomes, PCL and Lf-PCL-8 (CHETA/Lf ratio = 1:8, w/w) showed an improved performance in the uptake efficiency and in the cytotoxicity as well as much improved localization in the brain [142]. Taken together, these results encourage further investigation for the application of Lf-PCLs to treat other brain diseases.

Other authors investigated whether natural bLf or its different iron-saturated forms, as dietary supplements, were able to increase the anti-tumour activity of different recognized anticancer drugs [143]. In this study, bLf was added to the diet of mice that were then challenged with cancer cells and treated with chemotherapy. Results obtained demonstrated that tumours in holo-bLf-fed mice were totally eradicated with a single injection of known chemotherapy agents whereas apo-bLf (4% iron saturated) or native bLf (about 15% iron saturated) were ineffective. To be fully effective in eradicating tumours, iron-saturated bLf (holo-bLf) had to be administered to mice for more than two weeks before the chemotherapy, indicating that it functions as a competence factor. In particular holo-bLf decreased tumour vascularity and increased anti-tumour cytotoxicity, apoptosis and infiltration of leukocytes in tumours. Holo-bLf bound to intestinal epithelium and enhanced the production of cytokines within the intestine and tumour, as well as nitric oxide that are known to sensitize cancer to chemotherapy. These results that may seem paradoxical are related to the fact that holo-bLf can release iron and trigger an inflammatory reaction. Holo-bLf also restored peripheral blood cell numbers depleted by chemotherapy, thus defending mice from cancer [143].

A subsequent study, based on emerging nanotechnologies, has been carried out to further improving the bioavailability of holo-bLf to tumour sites by developing polymeric-ceramic nanocarriers (NCs) [144]. The authors validated the preclinical efficacies of novel NC oral formulations for the delivery of holo-bLf in colon cancer therapy. Further insights into the therapeutic application of lactoferrin encapsulated in NCs can be found in the review of Sabra and Agwa [145].

In summary, iron-saturated bLf is a powerful natural adjuvant and a fortifying agent capable of improving cancer chemotherapy. As already said, currently the extraction of Lf from cow’s milk and its use in various products represents an industrial reality and it is therefore likely that, in the future, the consumption of bLf containing dietary products could be suggested to inhibit or delay the onset of cancer.

## 8. Other Therapeutic Properties of Lactoferrin

There are many other potential uses of bLf for improving human health and some of them will be discussed below.

### 8.1. Lactoferrin and Obesity

Obesity represents a serious public health problem and is a strong predictor of chronic diseases. It is now recognized that the intestine and its commensal microflora play an important role in the development of chronic inflammation related to obesity. In fact, obesity and a diet rich in fats are associated with an alteration of the gut microbiota and an increase in intestinal permeability that allows the translocation of LPS into the circulation contributing to systemic inflammation. bLf has been used successfully in the prevention and treatment of obesity and inflammation by inducing the reduction of visceral fat, the neutralization of bacteria in the mucous membrane and the reduction of intestinal permeability.

Concerning the control of fat accumulation, it has been demonstrated that bLf oral administration during caloric restriction in mice was able to enhance weight loss and induced a significant reduction in the total fat pad weight and adipocyte size [146]. Moreover, it has been shown in a mice model with unrestricted food intake that bLf administration induced visceral fat reduction and affected mesenteric adipocytes and fatty acid metabolism in the liver, decreasing the size of mesenteric fat without modulating body weight [147].

Based on the observation that the intestinal commensal microflora plays an important role in the obesity control [148], Sun et al. [149] investigated the role of bLf in obesity as a prebiotic compound, demonstrating that oral administration of 100 mg/kg BW bLf for 12 weeks in high-fat diet induced obese mice was able to positively modulate gut microbiota, inhibited inflammation, reduced body weight and fat accumulation, regulated glucose metabolism and relieved liver steatosis. These results are in agreement with previous reports suggesting a healthy role for the supplementation of bLf in the prevention of metabolic complications related to obesity [150].

More recently, Xiong et al. [151] have confirmed the modulatory effects of bLf on lipid metabolism, however the regulatory mechanisms still remain unclear. Moreover, this study has been carried out on high-fat diet-induced obese C57BL/6J mice in which oral administration of bLf for 15 weeks significantly decreased fat tissue weight, visceral adiposity and hepatic lipid accumulation. These effects are probably due to the suppression of lipogenic gene expression and to the improvement of liver and epididymal adipose tissue inflammation.

The effect of bLf administration has also been studied in association with other safe and effective substances. For example, since bLf and metformin both exhibit beneficial effects on body weight management and fat accumulation, their effects, alone or in combination, on lipid accumulation and metabolism in mice fed with high fat diet has been also studied [152]. Results obtained showed that bLf and metformin, both alone or in combination, prevented high fat diet induced obesity and improved lipid metabolism. The actions bLf and metformin that significantly decreased body weight, waist circumference, Lee’s index and visceral fat but had no effect on liver weight were partially due to a key kinase regulating cellular energy homeostasis, the AMP-activated protein kinase.

Regarding the administration of bLf in humans, the effect of enteric-coated bLf was also studied in a randomised double-blind placebo-controlled trial. This human clinical trial also reported that eight-week bLf consumption decreased total adiposity and visceral fat accumulation in male and female subjects with abdominal obesity [153].

In conclusion, numerous studies have shown that dietary bLf consumption represents a favourable agent for the control of lipid accumulation however, prospective as well as mechanistic analyses are still necessary to explain the lipolytic role of bLf in the diet and its potential applications for the obesity treatment. Results of these preclinical studies are summarized in Table 2.

Lastly, an important aspect to consider is the relationships between adiposity and serum iron as hypoferremia could be either an actual iron deficiency or a functional iron deficiency mediated by inflammation. In particular, the link among iron levels, bLf and obesity needs further investigations. This is very important, as it is now clear that iron deficiency and obesity are closely linked [154]. It still remains to be clarified the role of bLf in the two aspects of the relationship between iron and obesity: What is the mechanism that leads to an imbalance of iron in the presence of excess adipose tissue and how iron participates in the pathogenesis linked to obesity.

### 8.2. Lactoferrin and Bone Metabolism

It is established that the bones and the immune system are closely connected. Both tissues possess common progenitors and produce common cytokines and inflammation and bone loss coexist in several diseases such as arthritis, osteoporosis and periodontitis.

It is also known that both bLf and hLf are anabolic factor for skeletal tissue as they are able to exert strong proliferative and anti-apoptotic actions in osteoblasts, and a reducing or even inhibitory effect on osteoclastogenesis [155,156,157,158]. Lactoferrin also stimulated proliferation of primary chondrocytes [156,157].

It has been demonstrated in vitro that, at physiological concentrations, bLf not only stimulates the proliferation of bone forming cells, osteoblasts and cartilage cells, but it is also an effective osteoblast survival factor. In addition, lactoferrin is able to decrease osteoclast in a murine bone marrow culture system as well as in rabbit mixed bone cell culture [155] and, concerning in vivo systems, local injection of bLf in adult mice resulted in increased calvary bone growth [159]. Subsequent research was conducted to evaluate the effects of oral administration of bLf on bone physiology in an osteopenic rat model (ovariectomized rats) [160]. The results of this study showed that bLf oral administration protected osteopenic rats against the ovariectomy-induced reduction of bone volume, trabecular number and thickness. bLf administration also prevented the elevation of trabecular separation and increased bone mineral density in osteopenic rats. Moreover, after bLf treatment, serum TNF-alpha and IL-6 production was suppressed and serum calcitonin increased [160]. Finally, it has been demonstrated that oral administration of bLf in ovariectomized rats strongly stimulated the bone healing following tibial fracture [161].

Lactoferrin, as mentioned above, is also one of the many defence proteins present in saliva where it exerts a defensive activity against both periodontal bacteria and inflammatory processes.

Concerning periodontitis, it has been shown that orally administered liposomal bLf in Wistar rats is an effective preventive and therapeutic agent in decreasing alveolar bone destruction [87] significantly inhibiting LPS-induced alveolar bone reabsorption without interrupting orthodontic tooth movement [162]. Results of this study suggest that liposomal bLf could represent a powerful preventive or therapeutic agent to control periodontal inflammation. In fact, when liposomal bLf was administered orally to periodontitis subjects, a significant improvement in probing depth and a considerable reduction in the production of LPS-induced cytokines from peripheral blood mononuclear cells were obtained [163]. To clarify the mechanism by which bLf is able to prevent LPS-induced osteoclastogenesis without interrupting tooth movement, the same authors investigated its effects on compressive stress (CS)-induced osteoclastogenesis in comparison with those on LPS-induced osteoclastogenesis via osteoblasts in vitro [164]. Results obtained demonstrated that bLf fights bone destruction associated with periodontitis without inhibiting bone remodelling by CS-loading suggesting that its oral administration could be highly beneficial for control of periodontitis in orthodontic patients [164].

All these studies have shown that bLf plays an important physiological role in bone growth and healing and exerts a therapeutic role in various bone diseases. We can therefore conclude that bLf can be considered a useful therapeutic agent both for bone regeneration and for destructive bone diseases.

### 8.3. Lactoferrin and Dry Eye Disease

As mentioned above, lactoferrin is secreted into tears by the lacrimal gland. Dry eye, a multifactorial disease causing visual disturbances and instability of the tear film with potential lesion and inflammation of the ocular surface, is a very common condition with a high prevalence among the elderly. Tear lactoferrin level is an indicator of lacrimal secretory function and correlated with the severity of conjunctivocorneal epithelial lesions in patients with primary, secondary and non-Sjögren’s syndrome dry eyes [165].

Kawashima et al. [166] studied in a mouse model whether oral administration of bLf was able to influence age-related tear dysfunction. The results obtained showed that lactoferrin, administered orally, can preserve the function of the lacrimal gland in elderly mice by mitigating oxidative damage and suppressing subsequent inflammation of the gland.

Concerning investigations on human subjects, it has been demonstrated that oral administration of bLf (270 mg/day for one month) represents an efficient treatment modality to improve tear stability and preserve ocular surface epithelium in dry eye patients with Sjögren’s syndrome. The authors attribute the tear function and ocular surface improvements to the suppression of inflammatory mediators by bLf [167]. More likely these effects of bLf on tear secretion are due to the combination of its direct action on the tear glands and the overall improvement of body metabolism.

Taken together these results suggest that the use of bLf as a dietary supplement may be a new and safe therapeutic alternative for patients with dry eye syndrome. Moreover, as it has been described in the section on bone metabolism, bLf could also prove useful in the prevention and therapy of other age-related diseases such as autoimmune, neurodegenerative and immune hypersensitivity disorders [168].

## 9. bLf on the Market

Large-scale preparation of bLf from cheese whey or skim milk has made this protein accessible commercialized health product for human and animals. The first important application of bLf in a commercial product was its supplementation in infant formula and has been subsequently used for the supplementation of various foods (such as probiotic foods to enhance the beneficial intestinal flora or functional foods to increase iron absorption), for skin care (in cosmetics as antioxidant) and oral care products (to provide oral hygiene), and as nutraceutical, to improve the immune system and to inhibit the inflammatory response. bLf is also used for the conservation and safety of food as it delays lipid oxidation [169] and inhibits microbial growth. The use of bLf in the food industry includes: Meat and wine industry, fat process and dairy industry [170]. The increased demand for natural foods has also increased the importance of natural inhibitors such as bLf. Therefore, the applications of bLf in the food sector are growing remarkably. In all these applications, bLf is expected to express its natural antioxidant, anti-inflammatory, immunomodulatory and anticancer properties.

## 10. Conclusions

Lactoferrin is an extremely adaptable protein that has been designated by natural selection to be a first-line defence in mammals. This key protein of natural immunity shows many kinds of marvellous biological activities in vitro and in vivo and helps us to defend against external aggressions both of infectious and non-infectious origin. In fact, being positively charged, it can bind numerous surface molecules or metal ions inducing the host’s immunomodulatory activation, which in turn affects both adaptive and innate immunity.

The focus of the present review was on several important health-promoting effects of this multi-functional nutraceutical protein although, given the growing array of applications of lactoferrin in the field of human health, coverage is certainly not complete.

Still, the (intentionally diverse) application domains here reviewed should substantiate the main message I meant to convey: This pleiotropic substance accompanies us and defends us throughout our life, from birth to old age, it is safe and is considered by the United States Food and Drug Administration as a GRAS product with no contraindications in patients of all ages. Besides, it represents an ideal nutraceutical product, cheaply produced from bovine milk, and numerous products containing bLf alone or in association with other nutraceuticals, supplements or probiotics are currently being commercialized.

Taken together, the evidence summarized in this review indicate that it would be advisable to embrace a more comprehensive and integrated approach to various diseases, whereby improvement in the patient’s quality of life, and even the clinical outcome, may be obtained by combining bLf with conventional therapies, as suggested by the studies examined here.

Such a perspective should motivate the collection of more data in order to improve our understanding of the protective role of bLf, in relation to both non-communicable diseases and infectious diseases.

I hope to have kindled the interest of the reader in the numerous and often interconnected beneficial activities of this surprisingly versatile milk protein.

## Figures and Tables

**Table 1:** Effect of bovine lactoferrin (bLf) in neonates: Clinical trials.

Infants	Study Design	Intervention	Main Results	References
VLBW neonates (<1000 g at birth)	Multicentre, double-blind, placebo-controlled, randomized trial	bLf (100 mg/d) or bLf + LGG 6.10^9^ CFU/d) or placebo.	bLf alone or in combination with LGG reduced the incidence of a first episode of late-onset sepsis in VLBW neonates.	[58]
VLBW neonates (<1500 g)	Multicentre, double-blind, placebo-controlled, randomized trial	bLf (100 mg/d), or bLf + LGG 6.10^9^ CFU/d) or placebo.	NEC or death incidence was significantly lower in groups bLf and bLf + LGG. No adverse effects or intolerances to treatment occurred.	[59]
VLBW (<1500 g) or <32 weeks neonates	Single-centre, double-blind, placebo-controlled, randomized trial	bLf (200 mg/d) or placebo, during hospitalization period	bLf prophylaxis decreased nosocomial sepsis episodes and increased Treg levels. Treg level increasing is suggested to be responsible for decreased sepsis.	[60]
VLBW (neonates <2000 g)	Placebo-controlled, double-blind, randomized trial	bLf 80–140 mg/kg/d or placebo for 4 weeks	bLf supplementation decreased the incidence of first episode of LOS.	[61]
Neonates, (<2500 g at birth)	Placebo-controlled, double-blind, randomized trial	bLf 200 mg/kg/d or placebo for 4 weeks	bLf supplementation decreased nosocomial sepsis episodes, especially in VLBW neonates.	[62]
Neonates <32 weeks or <1500 g	Placebo-controlled, double-blind, randomized trial	bLf (100 mg/d) or bLf + LGG 1.10^6^ CFU/d) or placebo.	bLf supplementation decreased the incidence of severe NEC.	[63]
VLBW preterm neonates	Multicentre, double-blind, placebo-controlled, randomized trial	bLf (100 mg/d) or bLf + LGG 1.10^6^ CFU/d) or placebo.	bLf supplementation, alone or in combination with LGG, reduced the risk for infections related to inhibitors of gastric acidity. bLf decreased the incidence of LOS and NEC.	[64]

LGG: *Lactobacillus rhamnosus* GG; VLBW: Very low birth weight; CFU: Colony-forming units; Tregs: T-regulatory cells; LOS: Late-onset sepsis; NEC: Necrotizing enterocolitis.

**Table 2:** Impact of bLf on the metabolic syndrome components or obesity: Preclinical studies.

Animal Species (Male)	Dose	Administration	Treatment Length	Outcomes	References
ICR mice	100 mg/kg	Gastric intubation	4 weeks	Reduced mesenteric fat tissue and hepatic lipid accumulation	[147]
High-fat diet induced obese C57BL/6J mice	(190 mL/kg) 100 mg/kg	Oral	7 weeks	Reduced body weight, obesity, adipose tissue (visceral, abdominal) and plasma glucose	[146]
High-fat diet induced obese C57BL/6J mice	100 mg/kg	Oral	12 weeks	Reduced body weight, fat accumulation, inflammation and relieve liver steatosis	[149]
High-fat diet induced obese C57BL/6J mice	100 mg/kg	Oral	15 weeks	Reduced weight gain, visceral adiposity, serum glucose, inflammation and improved hepatic steatosis	[151]
High-fat diet induced obese C57BL/6J mice	100 mg/kg	Oral	50 days	Ameliorated fatty liver formation, exerted beneficial effects on glucose tolerance and adipocyte tissue inflammation without interfering energy intake.	[150]
C57BL/6 mice	2 g/100 mL alone or in combination with metformin	Oral	12 weeks	Improved high-fat diet-induced obesity and lipid metabolism.	[152]
